# Commentary: A bacterial global regulator forms a prion

**DOI:** 10.3389/fmicb.2017.00620

**Published:** 2017-04-11

**Authors:** Giovanni Di Guardo

**Affiliations:** Faculty of Veterinary Medicine, University of TeramoTeramo, Italy

**Keywords:** *Clostridium botulinum*, prions, amyloid, bacterial inheritance, bacteria, yeasts, prokaryotes, eukaryotes

The recently reported identification, within the transcription terminator Rho of *Clostridium botulinum* (*Cb*-Rho), of a prion-like domain functionally similar to that of a yeast prion-forming protein, along with the amyloidogenicity conferred by it on *Cb*-Rho, represent findings of extraordinary scientific relevance (Yuan and Hochschild, 2017). In this respect, another recent study has shown that lactic acid, a common bacterial metabolite, is a powerful inducer in yeast cells of [*GAR*^+^], a prion-like genetic element allowing the simultaneous metabolism of glucose and other carbon sources (Garcia et al., 2016). Still noteworthy, while synthetic mammalian prions were successfully generated in *Escherichia coli* bacteria (Legname et al., 2004, 2005), the amyloid-ß peptide—a protein crucially involved in Alzheimer's disease pathogenesis—has been recently reported to bind and trap bacterial pathogens inside the brain, thereby behaving like a natural antibiotic (Kumar et al., 2016). Furthermore, the normal host's cellular prion protein (PrP^C^) has been also shown to play a pivotal role in *Brucella abortus* infection of murine macrophages, with no evidence of bacterial colonization nor replication in cells from PrP^C^-deficient mice (Aguzzi and Hardt, 2003; Watarai et al., 2003).

Although no doubts seem to exist that, based upon the results of the elegant work (Yuan and Hochschild, 2017) which is being addressed by the present commentary, *Cb*-Rho acts like a prion-like element of inheritance in bacteria, I do not feel entirely confident about the Authors' conclusion, “*suggesting that the emergence of prions predates the evolutionary split between eukaryotes and bacteria*” (Yuan and Hochschild, 2017). As a matter of fact, although this would appear to be absolutely plausible from a biological standpoint, prior evidence of similar, or related prion-like domains in Rho or Rho-like proteins from other *Clostridium* genus members should be obtained to justify the Authors' statement. In this respect, *Clostridium* (*C*.) *baratii, C. butyricum*, and *C. tetani*, which are phylogenetically related to *C. botulinum* (Collins and East, 1998), could represent valuable “first choices” for “comparative” investigations of this kind. Finally, the prion-driven, putative evolutionary links between bacterial and eukaryotic cells could gain additional insights from the study of mitochondria, ubiquitous cytoplasmic organelles derived from an alphaproteobacterial endosymbiont, which were acquired in the course of eukaryogenesis (Poole and Gribaldo, 2014).

## Author contributions

After having carefully read the recent Science article by Drs. Yuan and Hochschild, upon which this manuscript is commenting, the Author (GD) has autonomously and independently written the present Commentary.

### Conflict of interest statement

The author declares that the research was conducted in the absence of any commercial or financial relationships that could be construed as a potential conflict of interest.
